# Dynamic weight-bearing assessment of pain in knee osteoarthritis: construct validity, responsiveness, and interpretability in a research setting

**DOI:** 10.1186/s12955-016-0495-6

**Published:** 2016-06-16

**Authors:** Louise Klokker, Robin Christensen, Eva E. Wæhrens, Elisabeth Bandak, Cecilie Bartholdy, Henning Bliddal, Marius Henriksen

**Affiliations:** The Parker Institute, Bispebjerg & Frederiksberg Hospital, Nordre Fasanvej 57, 2000 Frederiksberg, Copenhagen Denmark; The Research Initiative for Activity studies and Occupational Therapy, Institute of Public Health, University of Southern Denmark, Odense, Denmark; Department of Physical and Occupational Therapy, Bispebjerg & Frederiksberg Hospital, Copenhagen, Denmark

**Keywords:** Outcome measure, Knee osteoarthritis, Pain, Activity

## Abstract

**Background:**

The Osteoarthritis Research Society International (OARSI) has suggested to asses pain after specific activities consistently in clinical trials on knee OA. The Dynamic weight-bearing Assessment of Pain (DAP) assesses pain during activity (30 s of performing repeated deep knee-bends from a standing position). The purpose of this study is to evaluate the construct validity, responsiveness, and interpretability of the DAP for knee osteoarthritis (OA).

**Methods:**

One-hundred participants with knee OA were tested twice each with the DAP, the Knee injury and Osteoarthritis Outcome Score (KOOS), six-minute-walk-test (6MWT), and 6-min-walk-test with subsequent pain rating (6MWTpain), and once with a transition questionnaire (TRANS-Q) for the patient-reported change in pain after 12 weeks of exercise. Construct validity (baseline-scores) and responsiveness (change-scores) were estimated by Spearman Correlation Coefficients. We hypothesized that no correlations would be excellent (<0.7) (divergent validity), except for the 6MWTpain (convergent validity). The TRANS-Q was used for interpreting the DAP change-scores in terms of responsiveness and Minimal Important Change (MIC).

**Results:**

Divergent validity with the KOOS subscales (*r* = −0.31 to–0.45) and the 6MWT (*r* = −0.25) was supported. Convergent validity with the 6MWTpain was not supported (*r* = 0.54). The DAP change-scores corresponded to patient-reported change in pain (TRANS-Q), while correlations with change-scores on the other instruments were <0.35. The MIC was 2.4 DAP points.

**Conclusions:**

The DAP possesses divergent validity compared to other instruments for knee OA, supporting the potential for this new way of assessing pain directly during activity. Importantly, the DAP change-scores correspond to patient-reported changes in pain, showing responsiveness. A change of 2.4 or more can be interpreted as clinically relevant. The DAP is a promising alternative to using ‘pain on walking’ as a clinical trial inclusion criterion/outcome.

## Background

The Dynamic weight-bearing Assessment of Pain (DAP) for knee osteoarthritis (OA) is a simple test designed to assess pain during performance of a specified activity in patients with knee OA [1, 2]. It is a simple performance test requiring repeated deep knee bends from a standing position for 30 s, with subsequent rating of pain on a 0–10 numeric rating scale (NRS), where 0 indicates ‘no pain’ and 10 ‘worst pain imaginable.’ The DAP follows current quality standards [3] to scientifically qualify a common clinical practice: asking patients about their pain during performance of a test. An elaborated description of the test has been published previously [2].

Application of the DAP is in line with The Osteoarthritis Research Society International (OARSI) suggestion for clinical trials on knee OA; *"Pain can be assessed after specific activities (*e.g.*, a walk test). If a pain assessment occurs after an activity then the study team should ensure consistency throughout the trial with the type and duration of activity as well as the timing of the pain assessment after the activity"* [4]. This consistency is best obtained through a standardized test such as the DAP.

The DAP was developed based on input both from patients (with knee OA) and health care professionals. Through focus groups, ‘weight-bearing deep knee bends’ was identified as a clinically important pain-provoking activity that would translate to a feasible instrument for ‘pain during activity’ by adding patient reports of pain [1]. The first version of the DAP had two scores: the pain rating, and the knee bend count. A study on reproducibility showed excellent properties of the pain score in a population of patients with knee OA (Intra-rater ICC = 0.93 [95 % CI 0.83 to 0.97], corresponding to a Standard Error of Measurement (SEM = of 0.70, and a Smallest Detectable Change (SDC) of 1.95; inter-rater ICC 0.91, [95 % CI 0.78 to 0.96], SEM 0.86, SDC 2.39), whereas the knee bend count did not reach acceptable level [2]. Consequently, the knee bend score was omitted and thus the second version of the DAP now consists of a single pain score. Simultaneously we clarified the construct assessed with the DAP to be ‘pain during activity’ instead of ‘the interaction between pain and function.’ The level of reliability is similar to that of pain ratings subsequent to other performance tests in patients with hip pain [5] and knee OA [6]. However, the validity, responsiveness, or interpretability of combined performance tests and pain ratings have not been evaluated.

We anticipate that the DAP assesses pain during performance of a specific weight-bearing activity in patients with knee OA — a construct that adds a new perspective to existing instruments. Our objectives were to evaluate the construct (divergent and convergent) validity, responsiveness, and interpretability of the DAP instrument.

## Methods

### Participants

This study was nested within an assessor- and participant-blinded randomized controlled trial comparing corticosteroid injection with placebo given 2 weeks prior to 12 weeks of supervised exercise in people with knee OA [7]. Inclusion criteria for the trial were: age 40 and over, symptomatic and radiologically verified diagnosed knee OA, ‘pain while walking on a flat surface’ of at least 4 on a 0–10 NRS, and a body mass index of 20 or more, but less than 35 kg/m^2^. Exclusion criteria included use of intra-articular corticosteroids in the knee or participation in physiotherapeutic exercise for knee OA within the last 3 months, or severe concomitant diseases. All participants in this study gave informed consent before enrolling in the hosting trial. Each participant received a copy of the consent.

### Instrumentation

#### Dynamic weight-bearing Assessment of Pain (DAP)

The DAP [2] is a simple performance test with an integrated pain score, designed to provide useful information for monitoring treatment progress and evaluating treatment effects in knee OA. The patient is asked to perform as many standing knee-bends as possible within 30 s. For each knee bend, the knees should reach close to 90° of flexion and full extension. This movement is supervised by the rater. The test score is self-reported pain intensity during knee bends on a 0–10 NRS reported immediately after the 30 s of knee-bends as the worst pain during the test. Thus, the pain intensity score is an assessment of pain during performance of a specific weight-bearing activity. The DAP takes 1–2 min to perform, including verbal instructions. Administering the test does not require any equipment beside a stopwatch/watch. The DAP was applied at baseline and at the end-of-treatment visit.

#### Knee injury and Osteoarthritis Outcome Score (KOOS)

The KOOS [8] is a questionnaire for assessing patient-reported symptoms. KOOS consists of 5 subscales assessing different constructs: Symptoms (7 items), Pain (9 items), Function in daily living (16 items), Function in sports and recreation (5 items), and knee-related Quality of Life (QoL) (4 items). Responses are given using Likert boxes, and each question is assigned a score from 0–4. A normalized score is calculated for each subscale ranging from 0 (extreme symptoms) to 100 (no symptoms). Reliability of KOOS in an OA population has been reported as acceptable [9]. The KOOS questionnaire was applied at baseline and at the end-of-treatment visit.

#### Six-minute-walk-test (6MWT)

The 6MWT [10] is a walking test measuring the total distance walked in 6 min. The distance is a surrogate measure of functional capacity and cardiovascular function, originally used for patients with heart and lung diseases. The 6MWT has shown acceptable test-retest reliability and responsiveness in knee OA populations [11, 12]; it was applied at baseline and at the end-of-treatment visit.

#### Pain after 6MWT (6MWTpain)

Pain rating on a 0–10 NRS immediately after the 6MWT (6MWTpain) is not a standardized test. However, similar ratings of pain subsequent to a performance test have been applied in other studies [5, 6]. The 6MWTpain was included to compare the DAP with this other assessment of pain during a performance test. The 6MWTpain was applied at baseline and at the end-of-treatment visit.

#### Transition questionnaire (TRANS-Q)

Transition ratings, or Global Perceived Effect scales, are recommended as a core outcome measure in chronic pain trials [13] and have been used as an external criterion to determine responsiveness [14] or Minimal Important Change (MIC) [3] of other measurement instruments. A transition questionnaire (TRANS-Q), modified from Jaschke et al. [15], was used for asking the participants about their experienced change in pain after the intervention with the question: “Did your knee pain change since you entered this project?” Response options were: “It is unchanged,” “It is better,” and “It is worse.” The ‘unchanged’ response is given a score of 0, and no further questions are asked. The responses “It is better” and “It is worse” bring up a seven-point scale, with scores spanning from −7 (worst) to +7 (best), respectively. For the purpose of this study, a clinically important change in pain was defined as a TRANS-Q score of at least 2 (+2: a little better; −2: a little worse). No change was defined as a TRANS-Q score of 0 (no change) or +1/-1 (Almost the same, hardly any better/worse at all). The transition questionnaire was implemented in the hosting trial after the trial commenced and therefore applicable to only a subset of the trial participants. The transition questionnaire was administered only at the end-of-treatment visit.

#### Kellgren & Lawrence

The Kellgren & Lawrence grading scale is used to assess radiographic severity of knee OA based on radiographic features; osteophytes, periarticular ossicles, narrowing of joint cartilage, sclerotic tissue, altered shape of bone ends. The scores are: 0 (no x-ray changes of OA), 1 (doubtful presence of OA), 2 (minimal presence of OA), 3 (moderate presence of OA), and 4 (severe presence of OA) [16]. X-rays were taken at baseline and at the end-of-treatment visit.

#### Ahlbäck classification

The Ahlbäck classification of radiographic knee OA of the tibiofemoral joint also assesses radiographic severity of OA and has five grades: 1 (joint space narrowing, <3 mm), 2 (joint space obliteration), 3 (minor bone attrition, 0-5 mm), 4 (moderate bone attrition, 5-10 mm), 5 (severe bone attrition, >10 mm) [17]. X-rays were taken at baseline and at the end-of-treatment visit.

### Statistical analysis

Analyses involving hypothesis testing for validity and responsiveness (i.e., the validity of a change score), and determination of the Minimal Important Change (MIC) to interpret change scores of the DAP were conducted adhering to the COnsensus-based Standards for the selection of healthMeasurement INstruments (COSMIN) methodology [3, 18].

For validation studies, a minimum sample size of 50 is recommended, but larger samples are preferred [3]. There is currently no consensus on standards for determining sample size in MIC studies. The statistical analyses were performed using SAS statistical software (version 9.3; SAS Institute Inc., Cary, NC, USA) and follow the COSMIN standards [3]. As no gold standard exists for the construct ‘pain during activity,’ validity and responsiveness were evaluated through hypothesis testing. The construct validity of the DAP was evaluated by Spearman Correlation Coefficients with the other outcome instruments using baseline scores. Likewise, the responsiveness of the DAP was estimated by Spearman Correlation Coefficients with the other outcome instruments using change scores (baseline to end-of-treatment). There is no consensus about the magnitude of correlations required for acceptable convergent or divergent validity [3, 19], indicating that similar or different constructs, respectively, are assessed by the two instruments being compared. As the DAP was expected to assess a composite construct containing aspects of the constructs assessed by the other instruments, some correlation was expected. Thus, relatively high correlation criteria were applied, for both validity and responsiveness in this study; r >0.7 for convergence and r <0.7 for divergence, based on the common application of 0.7 as cutoff [20]. Correlations below 0.2 were disregarded, as this is the critical point for a two-tailed 0.05 level of significance in an *n* = 100 sample (the sample of the hosting trial) [7].

Both the DAP and the 6MWTpain were expected to assess a construct of pain during activity (albeit two different activities). Thus, for both baseline and change scores, the DAP score correlations with the 6MWTpain score were hypothesized to be convergent. The 6MWT reflects a construct of physical capacity/cardiovascular function, whereas the subscales of the KOOS assess symptoms, pain, function in daily living, function in sports and recreation, and knee-related quality of life. Thus, for both baseline and change scores, the DAP score correlations with the 6MWT and the KOOS subscales scores were hypothesized to show divergence.

Responsiveness was further evaluated by patient-reported change (TRANS-Q), hypothesizing that the group that had experienced a change in pain would have a greater mean change in DAP scores than the group reporting no change in pain. Patient-reported change of pain (TRANS-Q) were also used as an external criterion to interpret the DAP change scores in terms of the MIC, which we defined as the optimal cutoff point on a Receiver Operating Characteristic (ROC) curve (i.e., the value for which the sum of misclassifications ([1 – sensitivity] + [1 – specificity]) is smallest. [3] The 95 % limit cutoff point is calculated as mean change + 1.645 * SD change of the group of participants who reported no change [3].

## Results

**Fig. 1:**
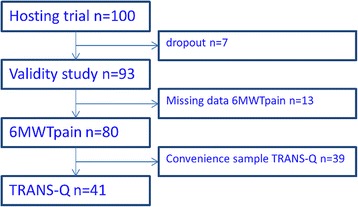
Numbers of participants enrolled in hosting trial (*n* = 100), included in the validity study (*n* = 93), completing the 6MWTpain (*n* = 80) and completing the TRANS-Q (*n* = 41)

As illustrated in Fig. 1, out of 100 participants included in the hosting trial, 93 participants who were still in the study by end of treatment were included in the present analysis of validity and responsiveness; though for the 6MWTpain data for further 13 participants were missing. A convenience subsample of 41 participants from the hosting trial was included in the analysis for responsiveness and interpretability using data from the TRANS-Q. Baseline characteristics for the 93 participants included in the validity study are presented in Table 1.

**Table 1:** Baseline characteristics (*n* = 93)

	*N* (%)	Mean	SD	Min	Q1	Median	Q3	Max
Gender, male	36 (38.0)							
Age		63.7	8.8	43.9	59.2	64.0	70.0	84.4
BMI		29.1	3.7	19.3	26.1	29.8	31.6	35.0
KL score (0–4, 0 = no OA)		2.8	0.8	1.0	2.0	3.0	3.0	4.0
Ahlback score (0–5, 0 = no OA)		1.0	1.1	0.0	0.0	1.0	1.0	4.0
X-ray, medial compartment most affected	83 (89.0)							
DAP (0–10, 0 = best)		3.8	2.2	0.0	2.0	4.0	5.0	9.0
6MWT distance		523	98	153	471	521	585	786
6MWTpain (0–10, 0 = best)		3.4	2.2	0.0	2.0	4.0	4.5	9.0
KOOSfunction (0–100, 100 = best)		62.0	16.3	16.2	50.0	64.7	73.5	94.1
KOOSQoL (0–100, 100 = best)		38.4	13.5	6.2	31.2	37.5	50.0	68.8
KOOSpain (0–100, 100 = best)		54.7	13.6	22.2	47.2	55.6	63.9	83.3
KOOSsport (0–100, 100 = best)		29.2	18.6	0.0	15.0	30.0	40.0	75.0
KOOSsymptoms (0–100, 100 = best)		58.3	17.3	14.3	42.9	60.7	71.4	92.9

*BMI* body mass index, *KL* Kellgren Lawrence, *DAP* dynamic weight-bearing assessment of pain, *6MWT* six minutes walking test, *6MWTpain* pain score (0–10 NRS) after 6MWT, *Qol* quality of life

### Validity

Our hypothesis about divergent validity was confirmed with correlations <0.7 between baseline-scores of the DAP and the 6MWT (*r* = −0.25), and the KOOS subscales (*r*-values ranging from −0.31 to −0.45) (Table 2). For baseline-scores of the DAP and the 6MWTpain, the correlation (*r* = 0.54) did not reach the criterion for convergence (r > 0.7), thus failing to support our hypothesis about convergent validity.

**Table 2:**
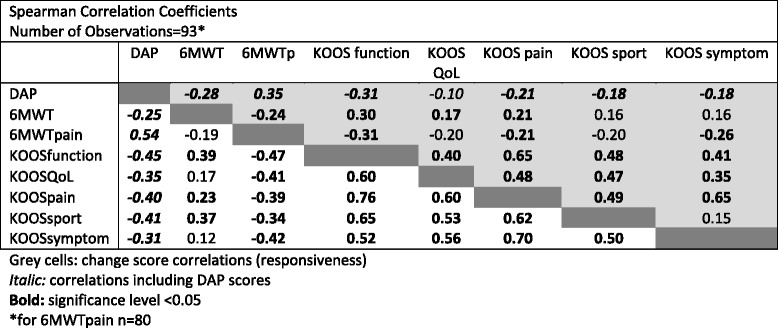
Correlation matrix (construct validity; baseline scores and responsiveness; change scores)

### Responsiveness

Our hypothesis about divergence was confirmed with correlations <0.7 between change-scores of the DAP and the 6MWT (*r* = −0.28) and the KOOS subscales (r-values ranging from −0.10 to–0.31) (Table 2). However, out hypothesis about convergence between the DAP and the 6MWTpain change-scores was not supported (*r* = 0.35).

Responsiveness of the DAP was supported, evaluated by patient-reported change in pain; the participants who had experienced a change (TRANS-Q rating of 2 or more) had higher DAP change scores (median change: −2.0 points) than the group reporting no change in pain (median change: −0.5 points; Table 3).

**Table 3:** Numbers of participants within each TRANS-Q response category and the corresponding DAP change median, range, mean, SD, and 95 % Confidence limits

		DAP change						
							95 % CI	
TRANS-Q (perceived change in pain)	*n* (*N* = 41)	Median	Min	Max	Mean	SD	Lower	Upper
7. A very great deal better	7	−3.0	−4.0	−1.0	−3.0	1.2	−4.9	−1.1
6. A great deal better	6	0.0	−2.0	2.0	−0.2	1.3	−2.4	2.0
5. A good deal better	4	−2.5	−5.0	−2.0	−3.0	1.4	−5.3	−0.7
4. Moderately better	6	−1.5	−8.0	0.0	−2.5	3.1	−7.6	2.6
3. Somewhat better	8	−2.0	−5.0	2.0	−1.9	2.2	−5.5	1.8
2. A little better	6	−0.5	−3.0	1.0	−0.7	1.6	−3.4	2.0
1. Almost the same, hardly any better at all	0							
0. The same	4	−0.5	−1.0	2.0	0.0	1.4	−2.3	2.3
−1. Almost the same, hardly any worse at all	0							
−2. A little worse	0							
−3. Somewhat worse	0							
−4. Moderately worse	0							
−5. A good deal worse	0							
−6. A great deal worse	0							
−7. A very great deal worse	0							
Improved (7 to 2)	37	−2.0	−8.0	2.0	−1.9	2.1	−5.3	1.6
no change (0)	4	−0.5	−1.0	2.0	0.0	1.4	−2.3	2.3

### Interpretability

**Fig. 2:**
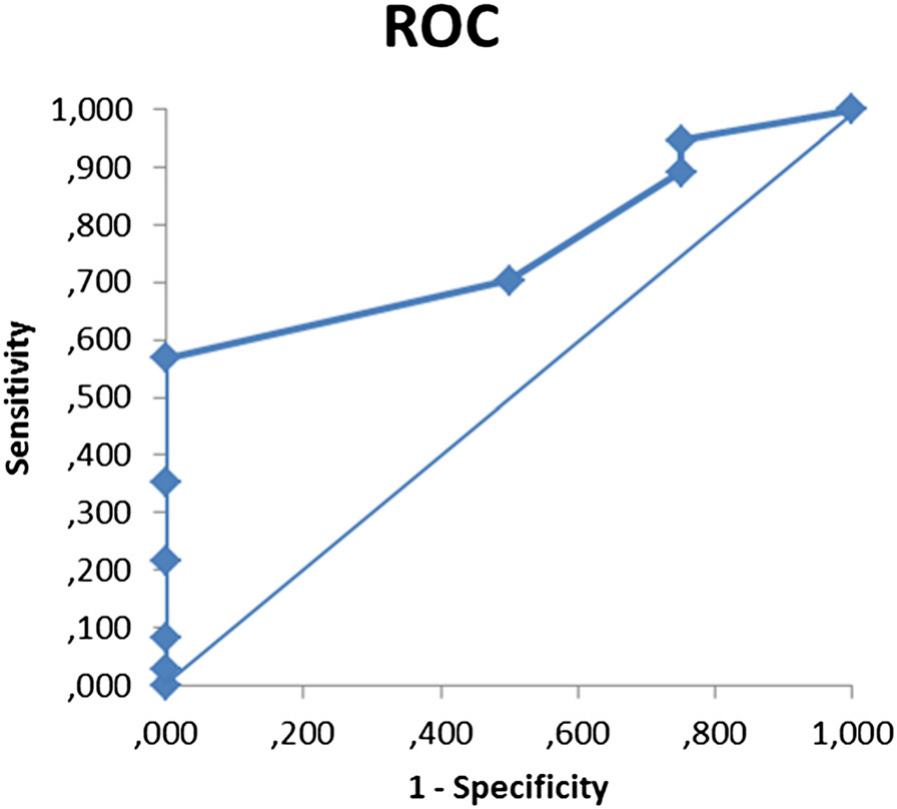
Receiver operating characteristic (ROC) curve for the various cutoff points for DAP change, using perceived change in pain as gold standard

The MIC was established to be 1.5 DAP-points using patient-reported change in pain (TRANS-Q of 2 or more) as gold standard, corresponding to a sensitivity of 0.57 and a specificity of 1.00 (Table 4 and Fig. 2). The 95 % limit cutoff point is 2.3 (calculated as 0.0 + 1.65*1.4), corresponding to the upper confidence limit in the group who experienced no change (Table 4). As the previously established SMD is 1.94 for intra-rater test, and 2.39 for inter-rater test [2], the MIC is changed to 2.4 DAP-points.

**Table 4:** Sensitivity and specificity for DAP change scores, using perceived change in pain as gold standard

DAP change^a^	Sensitivity	Specificity	1 - Specificity	1-Sensitivity	Sum [1-Sens + 1-Spec]
−9.00	0.000	1.000	0.000	1.000	1.000
−6.50	0.027	1.000	0.000	0.973	0.973
−4.50	0.081	1.000	0.000	0.919	0.919
−3.50	0.216	1.000	0.000	0.784	0.784
−2.50	0.351	1.000	0.000	0.649	0.649
**−1.50**	**0.568**	**1.000**	**0.000**	**0.432**	**0.432**
-.50	0.703	0.500	0.500	0.297	0.797
.50	0.892	0.250	0.750	0.108	0.858
1.50	0.946	0.250	0.750	0.054	0.804
3.00	1.000	0.000	1.000	0.000	1.000

total number of participants who perceived improvement (TRANS-Q score 1–7) = 37

total number of participants who perceived no change (TRANS-Q score 0) = 4

The sum of misclassification is used to determine the most appropriate cutoff score (marked with bold letters).

^a^negative number indicates a decrease in pain

## Discussion

These results suggest that the DAP has adequate properties for measuring a construct of pain during activity. This construct is not covered by widely used instruments for knee OA: the questionnaire KOOS and the performance test 6MWT, as confirmed by divergent validity. The correlation coefficient of 0.54 between the DAP and the 6MWTpain suggests that the constructs assessed with these two instruments are somewhat similar, although not fully convergent. The responsiveness of the DAP is adequate, shown by the different median DAP change scores in the group that reported change in pain by TRANS-Q and the group that reported no change, respectively. An improvement of 1.5 or more DAP points can be interpreted as clinically relevant, relating nicely to previous findings where the minimal clinically important difference in chronic musculoskeletal pain equals a reduction of 15.0 % on the 0–10 NRS [21]. However, as the smallest detectable change of the DAP was previously estimated to be 1.95 for intra-rater and 2.39 for inter-rater [2], the more conservatively estimated MIC of 2.4 (corresponding to the upper confidence limit of the mean DAP change in the group experiencing no change) should be used to inform clinical decisions for individual patients.

Ideally, evaluating the construct validity of the DAP should involve a comparison with a gold standard — another instrument that is proven to reliably assess pain during activity. As no such gold standard exists, we focused on the divergent aspect of construct validity, hypothesizing that the instruments focusing solely on pain (KOOSpain) or function (KOOSfunction, 6MWT) would not correlate highly (prespecified as r < 0.7) with the DAP. Furthermore we added a pain rating to an existing performance test (6MWTpain), expecting this addition to reflect a similar construct of pain during activity. A high correlation (prespecified as r > 0.7) with the 6MWTpain would thus support convergent validity. Although our assumption of divergent validity held true, the DAP and the 6MWTpain had lower correlations than expected, not supporting convergent validity. This finding may be explained by the differences between the DAP and 6MWTpain. Importantly, the 6MWTpain is not a standardized instrument and is not designed to incorporate a pain score. Further, the activities of knee bends versus 6 min of walking may not be equally pain provoking in a population of patients with knee OA. There are differences in the activities – knee bending and walking. During the performance of the two tests there were different responses from the patients; walking seemed to be more painful in the beginning, but then fading out, while the pain increased with more knee bends. This supports that the DAP assess a construct of weight-bearing activity that is relatively more pain provoking than 6 min of walking. The DAP is a simple test with a short duration and a focus on the pain intensity during performance of a specified activity. In the 6MWTpain, the distance walked is the main score, whereas the pain score can be considered a ‘nice-to-know’ add-on. Even though the exact construct assessed by the DAP cannot be described based on these results, it is suggested that the DAP assess a different construct than other commonly used instruments, but has the closest relation to the 6MWpain.

The correlation among change scores of the DAP and the 6MWTpain was low, potentially diluting the DAP responsiveness either due to poor qualities of the DAP or inappropriate selection of comparable instruments (lack of gold standard). However, the correspondence to patient-reported change in pain (TRANS-Q) may be seen as an even stronger indicator of actual change than other instruments show. Thus, we interpret the responsiveness of the DAP as adequate.

With a median DAP score of 4 (range 0–9) at baseline, most of the participants had relatively low pain levels, as also reflected by the other instruments. Nine participants reported to have no pain (DAP score 0 at baseline). No participants experienced worsening of their pain as assessed by TRANS-Q, and only four participants reported ‘no change’. This cause a limitation to the study, as only the MIC for improvement can be established, not for deterioration. Another limitation is constituted from our choice of anchor. Had the anchor been more specific, e.g. asking about pain during a weight-bearing activity, or more general, e.g. asking about wellbeing, the MIC may have been different. Likewise the TRANS-Q only gives information about the size of experienced change, not whether it is important. Thus, the choice of which change that is considered important was in this case not based on patient-reports. However, the final MIC of 2.4 is a conservative estimate, in this population corresponding to ‘a good deal better’ or more, which presumably reflects an important improvement.

It is a limitation to the DAP that not everyone experience pain during the test. This may reflect day-to-day variability of knee pain in patients with knee OA, or that pain occurs in various situations for the individual patient (at rest, walking, standing, kneeling etc.). Another possible explanation for the low pain level could be that the study population was eligible for pharmacological treatment and rehabilitation, thus not very severely affected. On the other hand, all participants did have both symptomatic and radiographic signs of knee OA, pointing at advanced knee OA. Including more severely affected patients (e.g., those eligible for surgery) could influence the results. However, DAP scores of 0 is not considered a ceiling effect because when there is no pain, there is no need for further discrimination on this parameter [3]. The DAP did not show signs of a floor effect either, as no participants reported ‘worst pain imaginable’ (DAP score 10). Whether this absence of floor and ceiling effects would hold true in a more severely affected population has not been established.

So far, the DAP has been tested by physiotherapists in a research setting only in a population of people with knee OA and mild to moderate symptoms, who mostly benefitted from the exercise intervention in a clinical trial. Testing beyond these limitations is ongoing.

## Conclusions

In conclusion, the DAP possesses divergent validity compared to currently used instruments for the assessment of knee OA, supporting the potential for this new way of assessing pain directly during activity. Importantly, the DAP change-scores correspond to patient-reported changes in pain, which proves adequate responsiveness. A change of 2.4 or more can be interpreted as clinically relevant. Hence, the DAP is a promising alternative to using ‘pain on walking’ as an inclusion criterion or as a clinical trial outcome.

## Abbreviations

6MWT, the 6-min-walk-test; 6MWTpain, the 6-min-walk-test with subsequent pain rating; BMI, body mass index; COSMIN, the COnsensus-based Standards for the selection of healthMeasurement INstruments; DAP, the dynamic weight-bearing assessment of pain; ICC, intraclass correlation coefficient; KL, Kellgren Lawrence; KOOS, the knee injury and osteoarthritis outcome score; MIC, minimal important change; NRS, numeric rating scale; OA, osteoarthritis; OARSI, The Osteoarthritis Research Society International; QoL, quality of life; ROC, receiver operating characteristic; SDC, smallest detectable change; SEM, standard error of measurement; TRANS-Q, transition questionnaire
